# Obituary

**Published:** 1893-06

**Authors:** 


### Obituary.

---

DR. CHARLES CARROLL LEE, of New York, died in hat city, May 11, 1893, of pleurisy, in the fifty-fifth year of his age. He was President of the Medical Society of the County of New York at the time of his death, Professor of Gynecology in the Post-Graduate Medical School, and Surgeon to St. Elizabeth's Hospital.

Dr. Lee was singularly well equipped, both by education and natural accomplishments, for the station in which he became such a shining light. As teacher and physician he had few equals and no superiors in his chosen field. His amiability of character and courteous demeanor on all occasions were such as to grapple a large group of friends to his soul with hooks of steel.

